# High Seroprevalence of *Toxocara* Infection among Mentally Retarded Patients in Hormozgan Province, Southern Iran

**DOI:** 10.1155/2021/2771837

**Published:** 2021-05-06

**Authors:** Mostafa Omidian, Mariye Diyaleh, Ali Pouryousef, Habibollah Turki, Fattaneh Mikaeili, Bahador Sarkari

**Affiliations:** ^1^Department of Parasitology and Mycology, School of Medicine, Shiraz University of Medical Sciences, Shiraz, Iran; ^2^Infectious and Tropical Diseases Research Center, Hormozgan Health Institute, Hormozgan University of Medical Sciences, Bandar Abbas, Iran; ^3^Basic Sciences in Infectious Diseases Research Center, Shiraz University of Medical Sciences, Shiraz, Iran

## Abstract

Mentally retarded individuals are more likely to become infected with soil-transmitted infections including toxocariasis. The current study aimed to determine the serostatus of toxocariasis among institutionalized mentally retarded individuals in Hormozgan Province, in the south of Iran. Subjects of the study were 117 mentally disabled individuals, including children and adults, maintained in a charity-based institution. Three millilitres of venous blood was taken from each subject. While sampling, demographic features of the subject were documented in a questionnaire. An ELISA system based on *Toxocara* larvae excretory-secretory antigens (TES) was utilized to detect anti-*Toxocara* IgG antibodies in the sera of the patients. The mean age of the subject was 27.6 (±12.31) years and consisted of 55 (47%) males and 62 (53%) females. Out of 117 cases, 33 (28.2%) were seropositive for toxocariasis. The seroprevalence was higher in females (37.1%) than males (18.2%), and the difference was statistically significant (*p* < 0.05). Out of 117 subjects, 76 (64.9%) had severe and 41 (35.1%) had profound mental retardation. Anti-*Toxocara* antibodies were detected in 18 (23.7%) patients with severe and 15 (35.6%) patients with profound mental retardation. The highest seroprevalence rate of toxocariasis (44.4%) was observed in the age group of ≤10 years followed by 21–30 years old (36.7%). No statistically significant association was seen between the age and also the duration of residency in the care center and seropositivity of toxocariasis (*p* > 0.05). The current study represents the high prevalence of toxocariasis in mentally retarded patients in Hormozgan Province, southern Iran. The elevated seroprevalence rate of toxocariasis in the current study indicates that these subjects constitute a high-risk group for *Toxocara* infection, which may be attributed to their behavioral patterns.

## 1. Introduction

Toxocariasis is one of the most important neglected soil-transmitted infections which is prevalent in tropical and subtropical areas of the world. *Toxocara canis* and *cati* (cosmopolitan roundworms of canine and feline) are recognized as causative organisms of human toxocariasis [1]. The most common ways in which humans become infected are consumption of vegetables and soil contaminated with *Toxocara* infectious eggs (eggs containing the second-stage larvae). Other less important means are consumption of contaminated water or undercooked/raw meat of paratenic hosts such as chicken, goat, sheep, and cattle [2]. After hatching eggs in the small intestine of the human, the infective larvae (L3) migrate to different tissues (especially liver, lungs, brain, and eyes) by blood circulation and cause two main syndromes: visceral and ocular, and less commonly neurological larva migrans syndrome. Human toxocariasis in most cases is asymptomatic, which is called covert toxocariasis [3]. Seroprevalence studies show a relatively high prevalence of human toxocariasis in different parts of the world [4–6]. In a recent study in Iran, the rate of *Toxocara* infection in cats and dogs, the main hosts of *Toxocara*, was reported to be 32.6% and 24.2%, respectively [7]. Moreover, the results of a meta-analysis study in Iran reported the rates of 0.84 to 29% for the seroprevalence of human toxocariasis in different areas of the country [8]. Despite several studies conducted in different parts of the country, studies conducted on high-risk groups in Iran are few [9–11]. Undoubtedly, one of these high-risk groups is the mentally retarded patients, who are mostly maintained in institutions with inadequate health facilities which increases their chances of getting infectious diseases including *Toxocara* infection [12].

The behavioral factors, e.g., sucking fingers, nail biting, and geophagia, along with inadequate personal hygiene in mentally retarded individuals lead to increasing the *Toxocara* infection in this high-risk group. Another risk factor for *Toxocara* infection is the geographical area where people live. South of Iran, due to its suitable climate conditions for the survival of *Toxocara* eggs in the environment, has provided favorable settings for people to be infected with this parasite [7].

The current study aimed to determine the serostatus of toxocariasis among institutionalized mentally retarded individuals in Hormozgan Province, in the south of Iran.

## 2. Methods

### 2.1. Sampling

This cross-sectional study was carried out on the mentally disabled subjects in the central rehabilitation center of Bandar Abbas, capital of Hormozgan Province in the southern Iran, from February to March 2021. Bandar Abbas is a port city on the southern coast of Iran (longitude 56°26′ E and latitude 27°17′ N), next to the Persian Gulf. The city is located on a flat area at 9 meters above the sea level. The city has a hot desert climate, where the maximum temperature in summer reaches 49°C; meanwhile, in winter, minimum temperature reaches 5°C above zero. The average annual precipitation is 171.4 mm (6.7 inches), and the average humidity is 65%.

The subject of the study was 117 mentally disabled individuals, including children and adults maintained in a care center, under the supervision of the country's welfare organization. The inclusion criteria were being the resident of the studied center and the likelihood of preparing a sample based on the individual condition, while the exclusion criteria were having fever and unwillingness of patients' parents to donate blood samples.

To provide the blood, 3 mL of fresh venous blood was collected from each subject; then, the serum was separated and kept at −20°C until use. Samples were transferred to the department of parasitology and mycology at Shiraz University of Medical Sciences, where they were evaluated by ELISA method. Demographic features such as age, sex, duration of residency, and severity of disability were documented in a questionnaire.

### 2.2. Serological Assay

An ELISA system based on excretory-secretory antigens (TES) of *Toxocara* larvae was utilized to detect anti-*Toxocara* IgG antibodies. TES was prepared as previously reported by Zibaei et al. [13]. In brief, *Toxocara* eggs were extracted from the female uteri worms. They were incubated at 25°C for 30 days in 2.5% formalin/ringer solution to become embryonated. RPMI medium was used to cultivate the second-stage larvae. The culture supernatant was concentrated; its protein content was measured and stored at −20°C until use.

To perform the ELISA, 96-well microplates (flat-bottom, Corning, USA) were coated with *Toxocara* antigen (5 *μ*g/mL) and incubated overnight at 4°C. The plates were washed five times by washing buffer (phosphate-buffered saline-Tween (PBST): 0.05% Tween 20 in PBS) via an automated ELISA plate washer. Skimmed milk (5% in PBST) was used to block the wells; after that, sera (1/100 dilution in PBST) were added (100 *μ*L/well) to the plate and incubated at room temperature for 1 hour. The washing procedure was repeated as before. Then, 100 *μ*L (1/4000 dilution in PBST) of peroxidase-labeled anti-human IgG was added to each well and incubated for 1 hour at room temperature. After washing as before, o-phenylenediamine dihydrochloride (OPD) solution (0.4 mg/mL OPD, 0.3% H_2_O_2_) was added, and the optical density (OD) was measured at 490 nm, using a microplate ELISA reader. By having negative and positive control samples in each run of the experiment, the cutoff value was calculated considering the mean of OD of negative samples plus two standard deviations (SD). In the end, the borderline and the positive samples were retested to confirm the results.

### 2.3. Statistical Analyses

SPSS v.22 was used for data analysis. Associations between toxocariasis seropositivity and ordinal and nominal variables were assessed with the Chi-squared test. *p* value was considered significant at the level of <0.05.

## 3. Results

A total of 117 mentally retarded patients were recruited in the present study. The mean age of the subjects was 27.6 (±12.31) years, ranged between 4 and 60 years old. The majority of cases (27.4%) belonged to the age group of 31–40 years. Subjects consisted of 55 (47%) males and 62 (53%) females.

Considering the calculated cutoff value (0.245), anti-*Toxocara* IgG antibodies were detected in sera of 33 out of 117 patients, indicating a 28.2% seroprevalence for toxocariasis. The seroprevalence was higher in females (37.1%) than males (18.2%), and the difference was statistically significant (*p* < 0.05). Out of 117 subjects, 76 (64.9%) had severe, and 41 (35.1%) had profound mental retardation. Anti-*Toxocara* antibodies were detected in 18 (23.7%) patients with severe mental retardation and 15 (35.6%) patients with profound mental retardation. The highest but nonsignificant seroprevalence rate of toxocariasis (44.4%) was observed in the age group of 0–10 years, followed by 21–30 years (36.7%). However, the association between the age group and seropositivity to toxocariasis was statistically insignificant (*p* > 0.05).

While the longest stay for the patients at the care center was more than 20 years, most of the subjects were maintained there for less than 5 years. The association between *Toxocara* seropositivity and duration of stay in the care center was statistically insignificant (*p* > 0.05).  Table 1 shows the features of mentally disabled patients and their association with seropositivity to toxocariasis in Hormozgan Province, southern Iran.

## 4. Discussion

Toxocariasis is an important zoonotic infection that is prevalent in both developing and developed countries. The disease is more prevalent in several high-risk groups, including children, mentally retarded patients, those people who own dogs or cats, and people who are living in a poor sanitary environment. Here, we determined the seroprevalence of *Toxocara* infection in mentally retarded patients, as a high-risk group for this infection, in Hormozgan Province, southern Iran, where 28.2% of the studied subjects were found to be positive for anti-*Toxocara* antibodies.

Patients with mental retardation are exposed to various infectious diseases, including parasitic infections, due to their care conditions and their unusual behaviors. Walking with bare feet in the caring institute, contact with soil, and difficulties in performing personal hygiene in our subjects were observed in this study.

Human epidemiological studies are difficult to compare since they have serious limitations due to different study designs and settings. Furthermore, confounding variables and bias are not adequately controlled. In a study carried out by Sharif et al. on mentally retarded children in northern Iran, the prevalence of intestinal parasites was reported to be 26.3% among the studied subjects [14]. In another study in the northern part of Iran, 173 mentally disabled individuals, aged 2–57 years, were evaluated for intestinal parasitic infection, where 51 (29.5%) of the cases were found to be infected with at least one parasite [15]. In Archelli et al.'s, study, the seroprevalence of toxocariasis in children under 3 years in Argentina was reported to be as high as 38.33% by ELISA and 45% by Western blot techniques [16]. In a study by Mohamed et al. on 150 mentally retarded patients in Egypt, anti-*Toxocara* antibodies were detected in 56.72% of the studied cases [17]. Kaplan et al. evaluated the seroprevalence of toxocariasis among 96 mentally retarded children and 85 healthy subjects, as the control group. Findings of the study revealed that the frequency of *Toxocara* infection is significantly higher in mentally retarded cases (18.8%) than in the healthy control group (7.1%) [18].

In a study performed on 5–15-year-old children in Bandar Abbas city (south of Iran), where the current study has been done, the seroprevalence rate of toxocariasis was reported to be 0.9% [11]. By comparing the findings of the present study with the study performed on healthy children in this region, it can be concluded that the rate of infection in mentally retarded patients is much higher than the prevalence reported in healthy children. This finding indicates that this particular group of people in the community is more susceptible to toxocariasis due to their exclusive living conditions and behaviors.

Toxocariasis has a tendency for infecting the central nervous system (CNS), which may result in neurological syndromes. Associations between mental disorders, including bipolar disorders or schizophrenia, and neurotropic parasitic infections including toxocariasis have been documented [19]. CNS involvement can lead to neuropsychiatric symptoms, encephalopathy, seizures, mental retardation, epilepsy, and neurodegeneration [20, 21]. Human neurotoxocariasis is relatively rare, yet it is not unreasonable to assume that the cause of mental retardation in some of our subjects in the present study is neurotoxocariasis, as myelitis, cerebellar vasculitis, space-occupying lesion, and mental retardation are accounted for the manifestation of human neurotoxocariasis [22].

Subjects of the current study were patients with severe as well as profound mental retardation. Patients with severe mental retardation have severe limitations in the areas of adaptive skills and intellectual functioning, characteristically exhibiting extensive deficits in sensorimotor skills through early childhood [23]. These patients usually experience severe restrictions in the areas of self-care, home living, social skills, community use, self-direction, and health. Individuals with severe mental retardation attain intelligence quotient (IQ) scores within the range of 20–25 to 35–40. Profound mental retardation is defined by the presence of meaningly subaverage common intellectual functioning along with significant restrictions in adaptive functioning existing before the age of 18 years. Patients with profound mental retardation obtain an IQ score below 20–25 [23]. In the current study, there was no significant difference between the two groups of patients in terms of *Toxocara* infection.

The mentally retarded subjects in the current study consisted of both children and adults whose ages ranged between 1 and 60 years. The rate of *Toxocara* infection is expected to increase with age, and this pattern has been reported in several studies [4, 16]. However, in the present study, no significant association was observed between age and seropositivity to toxocariasis. The reason for this might be that people with mental retardation are at a risk of acquiring the disease from an early age, and similar risky behaviors are seen in all age groups, both children and adults.

The findings of this study increase our knowledge about the infection rate of a soil-transmitted helminth in one of the high-risk groups in the community and indicate that the behavioral patterns of individuals may play an important role in the epidemiology of the disease. From a health point of view, the high prevalence of toxocariasis in mentally disabled individuals somehow indicates the unfavorable living conditions of these people, and it is necessary to take preventive measures in this regard, especially to prevent contact of these people with soil, to reduce the rate of infection in these people.

To the best of our knowledge, the present study is the first seroprevalence study of toxocariasis carried out on mentally retarded patients in Iran.

Limitations of this study include the low sample size, lack of information about the details of retardation of the subjects, and also the relatively high distribution of the age of the subjects.

## 5. Conclusions

The findings of the current study represent the high seroprevalence of toxocariasis in mentally retarded patients in southern Iran. Determination of the high seroprevalence rate of *Toxocara* infection indicates that these subjects constitute a risky group for *Toxocara* infection, and the high frequency of *Toxocara* infection may be attributed to their behavioral patterns.

## Figures and Tables

**Table 1:** Demographic features of the mentally retarded patients and relative seropositivity to *Toxocara* in Hormozgan Province, Southern Iran.

Characteristics	Frequency		Positive for anti-*Toxocara* antibodies		*p* value
	No.	Percent	No.	Percent
*Age*					0.302
≤10	9	7.7	4	44.4	
11–20	30	25.6	9	30	
21–30	30	25.9	11	36.7	
31–40	32	27.4	5	15.6	
>41	16	13.7	4	25	

*Sex*					
Male	55	47	10	18.2	0.026
Female	62	53	23	37.1	

*Retardation type*					
Severe	76	65	18	23.7	0.196
Profound	41	35	15	36.6	

*Duration of residency*					
≤5	44	37.6	14	31.8	
6–15	22	18.8	5	22.7	
11–15	24	20.5	4	16.7	0.337
16–20	10	8.5	5	50	
≥21	17	14.5	5	29.4	

## Data Availability

The datasets generated and analyzed during the current study are available in the table. The quantitative data used to support the findings of this study are available from the corresponding author upon request.
